# High thioredoxin-1 levels in rheumatoid arthritis patients diminish binding and signalling of the monoclonal antibody Tregalizumab

**DOI:** 10.1038/cti.2016.69

**Published:** 2016-12-23

**Authors:** Katharina Heim, Benjamin Dälken, Stefanie Faust, Faiza Rharbaoui, Andre Engling, Holger Wallmeier, Theodor Dingermann, Heinfried H Radeke, Jörg Schüttrumpf, Marcus Gutscher

**Affiliations:** 1 Biotest AG, Dreieich, Germany; 2 Condor Scientific Computing and Consulting, Sulzbach, Germany; 3 Institute of Pharmaceutical Biology, Goethe University Frankfurt, Frankfurt, Germany; 4 Institute of Pharmacology and Toxicology/ZAFES, Clinic of the Goethe University, Frankfurt, Germany

## Abstract

The humanized non-depleting anti-CD4 monoclonal antibody Tregalizumab (BT-061) is able to selectively activate the suppressive function of regulatory T cells and has been investigated up to phase IIb in clinical trials in patients suffering from rheumatoid arthritis (RA). A pharmacokinetic–pharmacodynamic model based on clinical data from RA and healthy volunteers, which used the cell surface CD4 downmodulation as marker of activity, confirmed a stronger effect in healthy volunteers compared with RA patients. We tried to understand this phenomenon and evaluated the influence of the small oxidoreductase thioredoxin-1 (Trx1). To counteract oxidative stress that is strongly associated with RA pathophysiology, the organism employs Trx1. Therefore, increased expression and secretion of Trx1 is found in the synovial fluid and plasma of RA patients. Moreover, the binding site of Tregalizumab is in close proximity to a disulphide bond in domain 2 (D2) of CD4, which is a known target for a reduction by oxidoreductase Trx1. With the experiments reported herein, we demonstrated that specific reduction of the D2 disulphide bond by Trx1 led to diminished binding of Tregalizumab to recombinant human soluble CD4 and membrane-bound CD4 on T cells. Moreover, we showed that this caused changes in the Tregalizumab-induced CD4 signalling pathway via the lymphocyte-specific protein tyrosine kinase p56^*Lck*^ and CD4 downmodulation. In summary, we provide evidence that high Trx1 levels in RA patients compared with healthy subjects are a potential reason for diminished binding of Tregalizumab to CD4-positive T cells and offer an explanation for the observed decreased CD4 downmodulation in RA patients in comparison to healthy subjects.

Rheumatoid arthritis (RA) is a common autoimmune disorder, which is characterized by chronic inflammation, bone and cartilage damage.^1, 2^ Autoimmune disorders are caused by an aberrant immunological function, including a failure of immunological self-tolerance leading to inflammations as seen in RA.^2, 3^ To control excessive immune responses regulatory T cells (Tregs) have an important role in immune homeostasis to maintain this self-tolerance.^4, 5^ Tregs represent a T-cell subset, which is able to suppress pathological immune response of other surrounding cells.^6^ Similar to other T cells, Tregs are initially developed in the thymus and express, among others, the interleukin-2 receptor alpha chain (CD25) and the glycoprotein CD4 molecule on their cell surface.^4, 7^ In the periphery, Tregs become activated upon encounter with an antigen presented on the major histocompatibility complex class II on antigen-presenting cells. They are recognized via the T-cell receptor, which associates the CD4 molecule in its complex.^7, 8^ CD4 consists of four extracellular domains (immunoglobulin-like (Ig) domains D1–D4), a transmembran domain and a cytoplasmic domain.^9, 10^ Three disulphide bridges are present in the CD4 molecule, of which the disulphide bond in D2 (between cysteines Cys155 and Cys184) is remarkable as this domain differs from the normal IgG structure.^11^ In the D2 domain, an intrasheet disulphide bridge substitutes an intersheet disulphide bridge, which is usually located in IgG domains.^11, 12^ Owing to its special geometry, a high dihedral strain energy and low calculated enthalpy D2 can be reduced selectively by the oxidoreductase thioredoxin-1 (Trx1).^13^ This reduction of the disulphide bridge has an important role in the HIV entry process, allowing the interaction with glycoprotein 120 (gp120) of HIV with the CD4 molecule.^14^ Trx1, which belongs to the thioredoxin family, is a small (12 kDa) oxidoreductase secreted by activated monocytes, lymphocytes and other immune cells,^15^ which exerts its function with help of thioredoxin reductase (TrxR) and NADPH in a disulphide exchange reaction^16^ (for a more detailed insight, the reader is referred to Lu and Holmgren^16^).

Many studies describe a significant association between RA and oxidative stress caused by reactive oxygen species^17, 18, 19^ and point out that Trx1 levels are augmented in patients suffering from RA^20, 21, 22, 23^ or other diseases, such as heart,^24^ lung^25^ and liver complaints,^26^ probably counteracting the increased oxidative stress in these diseases. Serum and plasma Trx1 levels were found to be elevated in RA compared with healthy subjects.^21, 22, 27^ Moreover, the synovial fluid of RA patients revealed higher Trx1 concentrations than those of osteoarthritis.^22, 27^ In addition, it was also demonstrated that RA disease activity correlated with elevated Trx1 plasma levels^22^ and Trx1 activity.^23^ The high concentration of Trx1 described for RA patients in particular^21^ might be associated with the hyporesponsiveness of T cells in RA patients.^20^

New strategies to tackle autoimmune reactions and to re-establish a good immunological balance are the enhancement of activity of CD4^+^ CD25^+^ Tregs. Therefore, anti-CD4 antibodies were investigated emphasizing CD4 as an interesting target.^28, 29^ Tregalizumab (BT-061) is a non-depleting IgG1 monoclonal antibody, which binds to a unique epitope of CD4 and represents the first humanized anti-CD4 monoclonal antibody that selectively induces Treg activation.^30^ As a new therapeutic approach, Tregalizumab has been investigated in eight clinical trials, including healthy subjects, RA and psoriasis patients. The antibody was investigated in RA to restore the functionality of defective Tregs and to enhance their suppressive capacity.^31^ Thereby, Tregalizumab exerts its agonistic function by binding to a unique, non-linear epitope on D2 of the human CD4 molecule and induces an intracellular signalling cascade involving the protein tyrosine kinase p56^*Lck*^.^30^ After administration of Tregalizumab, downmodulation of CD4 expression levels can be determined *in vitro* and *in vivo*, representing a marker for the antibody activity.^31, 32^ In the latest phase IIb trial (TREAT 2b, T-cell Regulating Arthritis Trial 2b, ClinicalTrials.gov Identifier: NCT01999192 and EudraCT No. 2013-000114-38) conducted with patients suffering from RA, the primary end point was not achieved. We describe in this manuscript experiments supporting a potential link between this observation and the reaction against the oxidative stress described in RA.

## Results

### Tregalizumab binds to CD4 in close proximity to the disulphide bridge in D2

**Figure 1:**
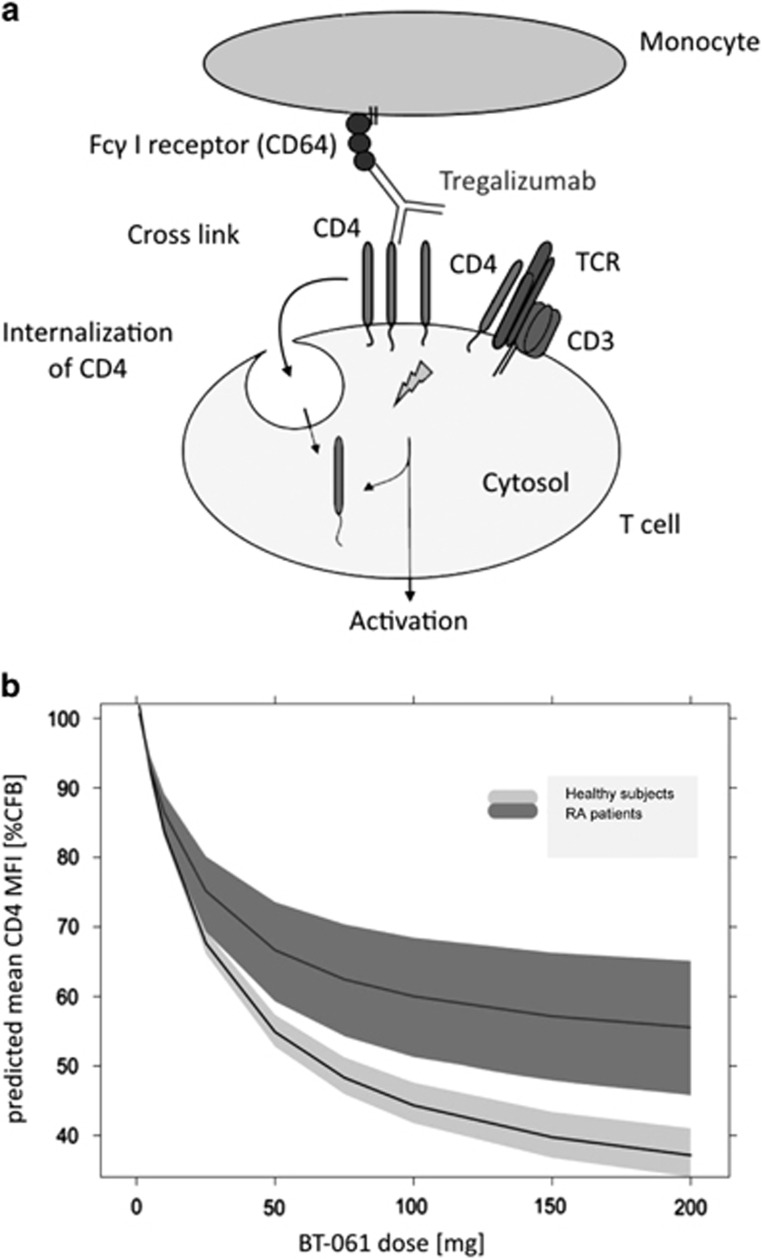
Tregalizumab mode of action and predicted CD4 modulation: (**a**) Tregalizumab (BT-061) binds to the CD4 receptor of T cells and is cross-linked via its Fc part by the Fcγ I receptor (CD64) on monocytes. This leads to a signal transduction into the cell and selective activation of regulatory Tregs. In addition, the CD4 receptor is internalized and turns off signalling. This CD4 downmodulation can be measured *in vitro* and *in vivo*. (**b**) The theoretical downmodulation of the cell surface protein CD4 after Tregalizumab administration as predicted by a pharmacokinetic–pharmacodynamic model is depicted after the first SC dosing (predicted mean values±the 95% confidence interval). The predicted CD4 downmodulation in healthy subjects (light curve) compared with patients with RA (dark curve) is shown.

The first step of the Tregalizumab mode-of-action is binding to the CD4 molecule via its Fab fragment followed by a cross-link with the Fcγ I receptor (CD64) on monocytes activating a signalling cascade.^33^ As outcome, Tregs are activated and CD4 molecules on regulatory and effector T cells bound by Tregalizumab are internalized (Figure 1a). In particular, a signal is transduced into the T cell via the CD4-associated protein tyrosine kinase p56^*Lck*^, leading ultimately to internalization of CD4 molecules and decline of surface expression. The declined CD4 expression refers to a decrease of CD4 on CD4-positive T cells that can be measured by flow cytometry *in vivo* and *in vitro*.^31^ Indeed, it was shown that Tregalizumab induces a decrease in the CD4 surface expression *in vitro* and in treated patients *in vivo*.^30, 31, 32^

**Figure 2:**
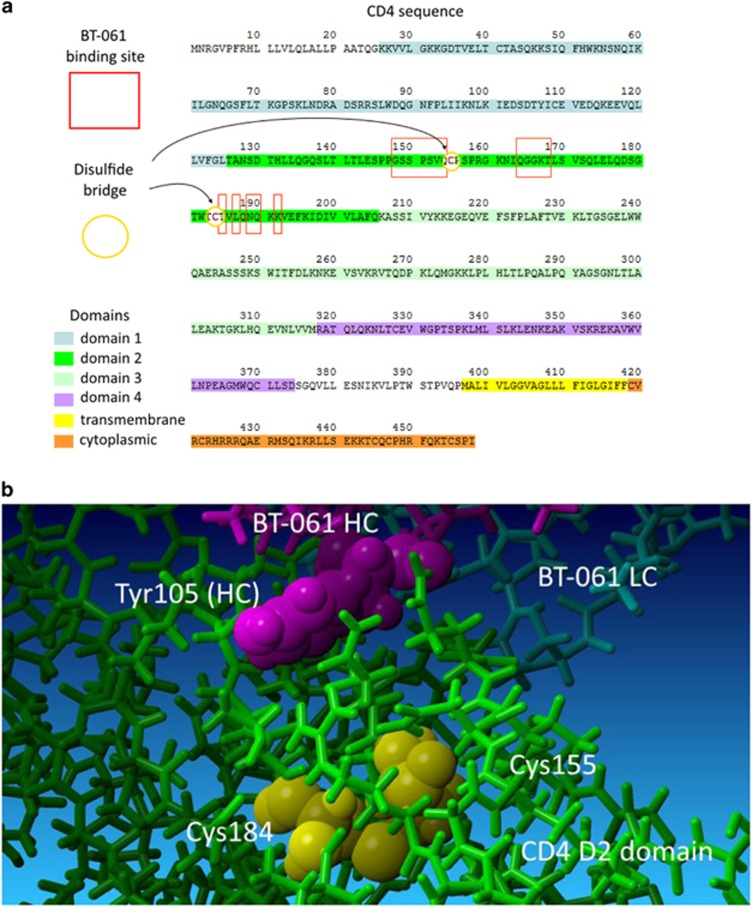
Tregalizumab binds to CD4 in close proximity to the disulphide bridge in D2. (**a**) Amino-acid sequence and domain structure of human CD4: The Tregalizumab-binding sites to CD4 are highlighted in red boxes (structure obtained by Proteros). The CD4 intramolecular disulphide bridge (Cys155–Cys184) in D2 is marked in red (yellow circle). (**b**) Crystal structure of the Tregalizumab–CD4 interaction. The D2 disulphide bond (marked in yellow) is in close proximity to the Tregalizumab-binding sites (Tregalizumab heavy chain in purple, light chain in cyan). Amino-acid tyrosine 105 (Tyr105) is displayed as space fill model and is important for the Tregalizumab–CD4 interaction.

For a better understanding of Tregalizumab's dose–response effects in patients, a pharmacokinetic–pharmacodynamic model was developed using the downmodulation of the CD4 molecule on the T-cell surface as pharmacodynamic marker (Rharbaoui *et al.*, manuscript in preparation).^32^ The pharmacokinetic–pharmacodynamic model based on clinical data from RA and healthy volunteer trials surprisingly showed higher CD4 downmodulation in healthy subjects compared with RA patients (Figure 1b). Looking for a possible explanation of this observation, we went back to the Tregalizumab–CD4 interaction. Protein crystallography revealed that Tregalizumab binds to D2 of CD4 to a conformational epitope that is not recognized by other anti-CD4 monoclonal antibodies.^30^ The epitope is close to an intramolecular disulphide bridge (Figure 2a). Distances from Cys155 and Cys184 to Tregalizumab's tyrosine105 (Tyr105) carbon atoms were calculated to be 7.0–11.0 Å. As this disulphide bond between cysteines Cys155 and Cys184 is in very close proximity to the binding interface of Tregalizumab to CD4 (Figure 2b), it is likely to influence the binding characteristics between both entities. This disulphide bridge has been described to be selectively reduced by Trx1.^13^ To examine whether reduction of this disulphide bridge may be responsible for higher CD4 downmodulation in healthy subjects compared with RA patients, we further examined the role of Trx1 on Tregalizumab binding to CD4 and the role of Trx1 in RA in general.

### Trx1 is able to reduce recombinant human soluble CD4

First, we demonstrated that reduced Trx1, as well as the Trx1/TrxR system, are able to reduce recombinant human soluble CD4 (rh sCD4) using rh sCD4 together with Trx1 or dithiotreitol (DTT) as a control. The Trx1 physiological system (Trx1/TrxR and NADPH) was also able to reduce rh sCD4 (Supplementary Figure S1). This is in accordance with results shown by Matthias *et al.*^13^

### Treatment with Trx1 diminishes Tregalizumab binding to CD4

Next, we elaborated whether reduction of rh sCD4 by Trx1 impacts the Tregalizumab–CD4 interaction by using a well-established electrochemiluminescence enzyme-linked immunosorbent assay (MSD system). Binding of Tregalizumab to Trx1-treated rh sCD4 was compared with untreated rh sCD4 control, which was set to 100%.

**Figure 3:**
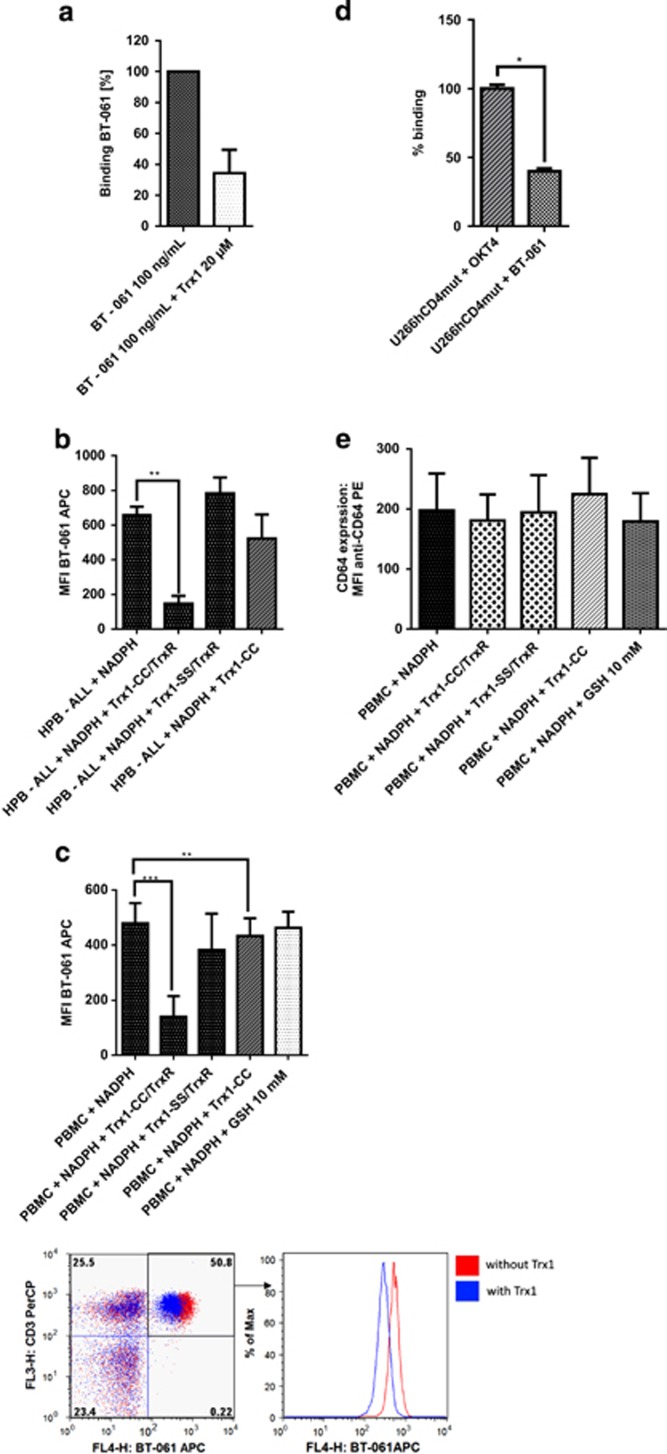
Trx1 diminishes binding of Tregalizumab to recombinant CD4 and CD4-positive cell lines. (**a**) Recombinant human sCD4 was incubated with 20 μM Trx1 for 30±5 min. After three washing steps, binding of Tregalizumab to rh sCD4 was measured using an electrochemiluminescence enzyme-linked immunosorbent assay. Data represent results from three independent experiments measured in triplicate. Error bars indicate s.d. *P*-values were calculated by means of Wilcoxon matched-pairs signed-rank test. (**b**) CD4-positive T-cell leukaemia cells (HPB-ALL) were incubated with 5 μM Trx1, 150 nM thioredoxin reductase and 1 mM NADPH for 15±2 h overnight. Binding of Tregalizumab to CD4 was measured using flow cytometry. The redox inactive thioredoxin1-SS-mutant (Trx1-SS) served as negative control. MFI of BT-061 APC is shown. Data represent results from four independent experiments performed in duplicates. Error bars indicate s.d. *P*-values were calculated by means of a paired *t*-test as the data are normally distributed. (**c**) Impact of the Trx1 system on binding of Tregalizumab to isolated PBMCs expressing CD4. PBMCs were incubated with 5 μM Trx1, 150 nM thioredoxin reductase and 1 mM NADPH overnight. The inactive mutant Trx1-SS served as negative control. Data represent results from five independent experiments performed in duplicates. Error bars indicate s.d. *P*-values were calculated by means of paired *t*-test as the data are normally distributed. The gating strategy is furthermore illustrated: lymphocytes were selected according to cell size and granularity (FSC/SSC) and the CD4 binding of Tregalizumab was determined in the CD3^+^ CD4^+^ gated cells. A representative dot plot (lower left) and histogram (lower right) of CD3^+^ CD4^+^ (BT-061^+^) cells is depicted with (blue) and without (red) Trx1/TrxR preincubation. (**d**) Comparison of Tregalizumab and OKT4 according to their binding to the mutated Cys/Ala D2 CD4 variant (hCD4 mut). MFI values of OKT4 were set to 100% and binding of BT-061 was calculated. Data represent results from two independent experiments performed in triplicate. Error bars indicate s.d. *P*-values were calculated by means of Wilcoxon matched-pairs signed-rank test. (**e**) PBMCs were incubated with either 5 μM Trx/150 nM TrxR/1 mM NADPH or 10 mM GSH/1 mM NADPH for 15±2 h overnight. After three washing steps, binding of fluorochrome-labelled anti-CD64 antibody was measured via flow cytometry (*n*=5, duplicates). Monocytes were gated according to their size and granularity (FSC/SSC). MFI of anti-CD64 PE is shown. Error bars indicate s.d. *P*-values were calculated by means of paired *t*-test as the data are normally distributed. Significance is shown as *P*-value: *P<0.05, **P<0.01, ***P<0.001.

As shown in Figure 3a, binding of Tregalizumab to rh sCD4 was decreased to 34.3±15.2% after pretreatment with 20 μM Trx1.

Next, the influence on binding of Tregalizumab to cell surface CD4 was assessed using the CD4-positive HPB-ALL cells under physiological conditions together with the Trx1/TrxR system.

HPB-ALL cells incubated with 150 nM TrxR plus 5 μM Trx1 overnight resulted in a significantly reduced binding of Tregalizumab from 661±91 to 147±45 mean fluorescence intensity (MFI) units of the BT-061 allophycocyanin (APC) staining (*P*=0.0041). This corresponds to a reduction of binding of 78% compared with the non-treated control (Figure 3b).

Incubation with 5 μM Trx1 alone without reductase displayed a less pronounced reduction of Tregalizumab binding. This is assumed as Trx1 without the reductase is not recycled and can facilitate the disulphide reduction process only once per molecule. As expected, the inactive control Trx1-SS was not able to reduce the Tregalizumab binding to CD4.

These results were confirmed using peripheral blood mononuclear cells (PBMCs) isolated from healthy subjects (Figure 3c). Pretreatment of PBMCs with Trx1/TrxR resulted in a significant (*P*=0.0007) decrease of 71% of Tregalizumab binding to CD4^+^ cells compared with untreated cells (from 479±73 to 140±75 MFI of the BT-061 APC staining), whereas the inactive mutant showed no significant decline of Tregalizumab binding. Preincubation of PMBC with Trx1 without reductase also revealed a less pronounced diminished binding of Tregalizumab comparable to the result observed on HPB-ALL cells.

The effect of Trx1 on binding of Tregalizumab to CD4 could also be confirmed using CD4-positive monocytes present in the PBMCs (data not shown).

Beside Trx1, other molecules are able to modulate the redox potential in the blood, such as glutathione (GSH), which represents a physiologically relevant reducing agent.^34^ To investigate a potential influence of GSH on the stability of the CD4 disulphide bridges and the interaction between Tregalizumab and CD4, further binding experiments were performed. Even when using GSH concentrations up to 10 mM, no significant effect on binding of Tregalizumab to CD4^+^ cells from PBMCs was observed (Figure 3c).

These results demonstrate that recombinant and cellular CD4 can be selectively reduced by Trx1, and this reduction diminishes the binding of Tregalizumab to the receptor. Other physiologically relevant reducing agents such as GSH do not influence binding of Tregalizumab to CD4.

### The D2 disulphide bond impacts Tregalizumab binding to CD4-positive cells

In order to confirm that the disulphide bond in D2 between cysteines Cys155 and Cys184 directly impacts Tregalizumab binding to CD4, a mutant of CD4 lacking both cysteines was constructed. CD4-negative U266B1 cells were transfected with CD4 variants, which are not able to form a disulphide bridge, as the corresponding cysteines Cys155 and Cys184 were replaced by alanine. Cells which were transfected with wild-type human CD4 served as control. The expression levels of the two CD4 constructs were different may be due to folding difficulties of the mutated CD4 as a result of the cysteine exchange. To compensate the different CD4 expression levels, the antibody OKT4, which binds to domain D3 of the CD4 molecule, was used for normalization. In comparison to OKT4, binding of Tregalizumab was decreased by 60% in the cells transfected with mutated CD4 (Figure 3d). This result further confirmed that the D2 disulphide bridge, which can be selectively reduced by Trx1, is crucial for binding of Tregalizumab to CD4.

### Trx1 has no influence on CD64 expression on monocytes

To test a potential influence on CD64 binding of Tregalizumab, PBMCs encompassing monocytes were incubated with the Trx1/TrxR system and the CD64 binding on monocytes was determined by flow cytometry using a specific anti-CD64 antibody. Overall, no significant difference in CD64 expression was observed after Trx1 pretreatment or GSH preincubation, respectively (Figure 3e).

### Trx1 influences Tregalizumab-induced CD4 signalling

To assess how the signal transduction induced by Tregalizumab is influenced by Trx1 and its physiological partners, phosphorylation of p56^*Lck*^ and zeta-chain-associated protein kinase 70 (ZAP-70) was analysed. In former studies, no significant differences in phosphorylation patterns of Tregs and effector T cells were observed after Tregalizumab stimulation.^30^ Hence, total CD4^+^ T cells isolated from PBMCs were analysed.

**Figure 4:**
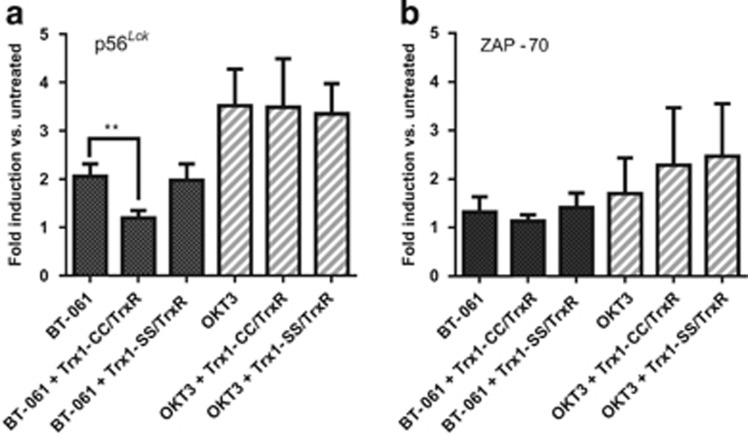
Impact of Trx1/TrxR on T-cell receptor signalling. A total of 10^5^ CD4^+^ cells were isolated from PBMCs and incubated overnight with 150 nM TrxR and 1 mM NADPH and either 5 μM active Trx1-CC or the inactive mutant Trx1-SS. Untreated cells were used as negative control. After the washing procedure, CD4^+^ cells were stimulated with either Tregalizumab (BT-061) or OKT3. Both antibodies were cross-linked with anti-human IgG (BT-061) or anti-murine IgG (OKT3). All data represent results from four independent experiments performed in duplicates. (**a**) Phosphorylation of lymphocyte-specific protein tyrosine kinase p56^*Lck*^ (pY505) was measured after intracellular staining by flow cytometry using anti-lck(pY505) antibody. (**b**) ZAP-70 phosphorylation was determined after intracellular staining by flow cytometry with anti-ZAP-70 (Y319)/Syk (Y352) antibody. Error bars indicate s.d. *P*-values were calculated by means of paired *t*-test as the data are normally distributed. Significance is shown as *P*-value: **P<0.01.

Phosphorylation of human p56^*Lck*^ after stimulation of cells with cross-linked Tregalizumab in the presence or absence of Trx1/TrxR was analysed by flow cytometry using a phosphorylation-specific antibody against p56^*Lck*^. Preincubation of CD4^+^ cells with Trx1/TrxR resulted in a significant decrease of phosphorylation (1.2±0.2-fold induction of phosphorylation, *P*=0.0057) compared with the Trx1 untreated control (2.1±0.3-fold induction of phosphorylation) and the inactive Trx1-SS control with TrxR (2.1±0.5-fold induction of phosphorylation; Figure 4a).

As a positive control for T-cell signalling, the CD3 antibody OKT3 was used. Stimulation of CD4^+^ cells with OKT3 revealed an increase in phosphorylation (3.5±0.8-fold induction of phosphorylation). Preincubation with Trx1 or Trx1-SS showed no significant change on OKT3-mediated phosphorylation (3.5±1.0- and 3.3±0.6-fold induction of p56^*Lck*^ phosphorylation, respectively). In contrast to p56^*Lck*^, pretreatment with active Trx1 showed no significant impact on ZAP-70 phosphorylation (Figure 4b).

Therefore, it can be concluded that reduction of the CD4 D2 disulphide bridge by Trx1 negatively impacts binding of Tregalizumab, which also affects the signalling pathway via phosphorylation of p56^*Lck*^, whereas ZAP-70 phosphorylation after CD3 stimulation is not impacted by the small oxidoreductase.

### Binding of Tregalizumab is decreased in RA patients compared with healthy subjects in clinical trials

Having established the effect of reduction of the D2 disulphide bond on Tregalizumab binding to CD4 *in vitro*, we evaluated whether the increased levels of Trx1 in RA patients lead to the same effect *in vivo*. Data from two clinical studies were analysed retrospectively concerning the binding of Tregalizumab to CD4. In total, results of 127 RA patients (Biotest study 979, EudraCT: 2010-018485-24) and 35 healthy subjects (Biotest study 985, EudraCT: 2011-004956-20) were evaluated. Relative Tregalizumab binding in relation to the total CD4 molecules on T cells was analysed.

**Figure 5:**
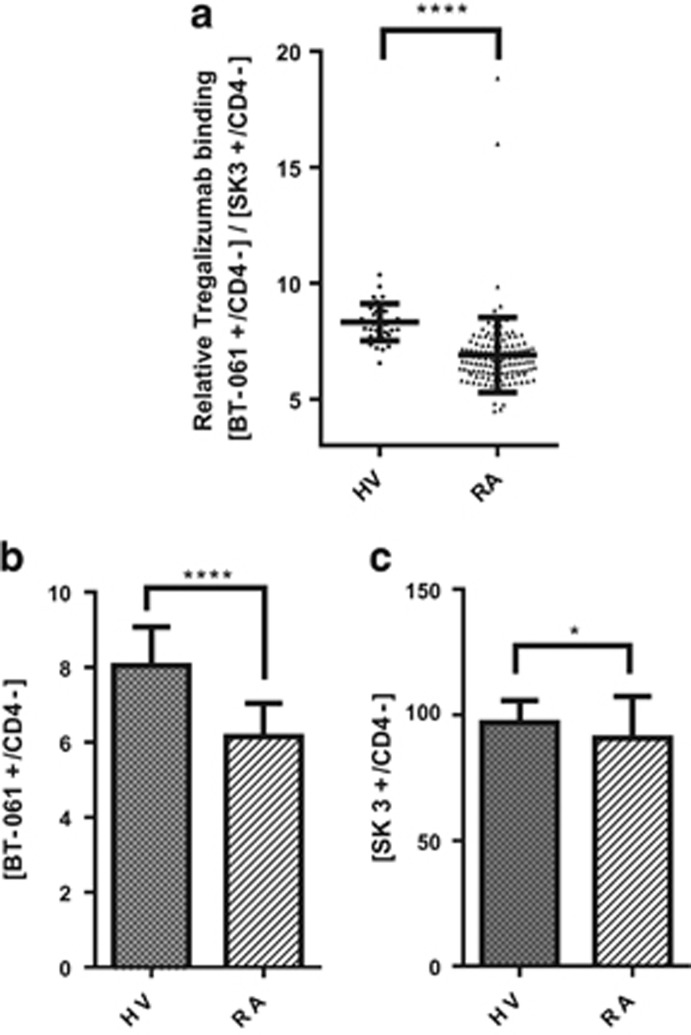
Tregalizumab binding to CD4 is lower in RA patients compared with healthy subjects. Data from two clinical studies (Biotest RA Study 979, EudraCT: 2010-018485-24; Biotest healthy volunteer Study 985, EudraCT: 2011-004956-20) were analysed retrospectively relating to binding of Tregalizumab to CD4. Binding of Tregalizumab to CD4-positive T cells was measured by flow cytometry using non-competing fluorochrome-labelled anti-CD4 antibodies (BT-061-FITC and SK3-PE). Thirty-five healthy subjects (Biotest study 985) and 127 RA patients (Biotest study 979) were analysed by the same central laboratory using the same assay system. The MFI values of BT-061-FITC and SK3-PE were used to calculate the relative binding of BT-061 in relation to the total CD4 expression (SK3 antibody). (**a**) Population-related relative binding of Tregalizumab in healthy volunteers (HV) and RA (RA) patients is depicted to show distribution of individual responses. (**b**) Mean relative binding of BT-061 to CD4 is depicted as a bar graph. Error bars indicate s.d. *P*-values were calculated by means of unpaired *t*-test as the data are normally distributed. (**c**) Mean relative binding of anti-CD4 antibody SK3 to CD4 is shown as a bar graph. Error bars indicate s.d. *P*-values were calculated by means of Mann–Whitney test. Significance is shown as *P*-value: *P<0.05, ****P<0.0001.

As shown in Figure 5a, relative binding of Tregalizumab (ratio of MFIs between BT-061 and the non-competing anti-CD4 antibody SK3) in RA patients is significantly (*P*<0.0001) lower (6.9±1.6, *n*=127) compared with healthy subjects (8.3±0.8, *n*=35). The observed difference in binding is due to the difference in mean binding of Tregalizumab to CD4 ([BT-061^+^/CD4^−^]), which is significantly lower in RA patients compared with healthy subjects (Figure 5b), whereas the overall CD4 expression ([CD4^+^/CD4^−^]) in general is comparable between RA patients and healthy subjects (Figure 5c).

## Discussion

The literature describes a strong connection between oxidative stress and RA disease, while our results demonstrated an impaired binding and signalling of Tregalizumab (BT-061) in this population. The work presented in this manuscript aimed to investigate a potential link between those observations.

Oxidative stress is considered to be a possible elicitor in the development and disease activity of RA^35^ and other diseases, such as metabolic disorder.^36^ One possibility for the organisms to counteract high levels of oxidative metabolites is to increase the level of the oxidoreductase Trx1. It represents one of the major players in redox processes for maintaining redox homeostasis,^16^ as it is able to reduce oxidized proteins. So numerous studies have investigated the relationship between Trx1 and the pathogenesis of RA.^20, 21, 22, 23^ They revealed that Trx1 protein and activity levels were augmented in patients suffering from RA.^20, 21, 22, 23, 27^ The literature observations on elevated Trx1 levels in plasma of RA patients were confirmed by our own analyses (data not shown).

Starting with the unexpected differences in predicted CD4 downmodulation between RA patients and healthy subjects, we were able to demonstrate that the disulphide bridge in D2 of the CD4 molecule, which can be selectively reduced by Trx1,^13^ is important for the Tregalizumab–CD4 interaction. We demonstrated that Trx1 reduces this disulphide bridge, which leads to a diminished *in vitro* binding of the Tregalizumab to its target. This could be verified with rh sCD4 in electrochemiluminescence experiments as well as with cell surface CD4 on T-leukaemia cell lines and primary cells analysed by flow cytometry. The CD4 molecule includes a disulphide bridge in D2 representing a so called −RHS staple and has a major role in other physiological processes owing to its allosteric properties.^37^ For example, dimerization of CD4 is important for the co-receptor function of CD4, which is induced by Trx1.^38, 39^ Apart from this fact, the HIV entry process is closely linked to redox changes in the D2 disulphide bond, implicating a relevant role of this molecule.^13, 14, 40, 41^ Regarding Tregalizumab, transfection experiments also emphasized the importance of this disulphide bridge in particular, as CD4 variants lacking the D2 disulphide bond showed a reduced binding of the antibody.

The pharmacological effect of CD4 downmodulation can only occur when the Fc part of Tregalizumab is cross-linked with the Fc-gamma receptor 1 (FcγRI, CD64) on monocytes.^33^ It could be possible that the higher CD4 downmodulation in healthy subjects compared with RA patients is caused either by a decreased binding of Tregalizumab's Fab part to CD4, by diminished expression or by modification of the FcγRI CD64. As the expression of CD64 is not influenced by Trx1 and no disulphide bridges are described for the CD64 receptor,^42^ Trx1 can only influence binding of Tregalizumab to CD4 and not to the CD64 receptor on monocytes. Binding of Tregalizumab to CD4^+^ monocytes was assessed (data not shown) and confirmed the assumption that binding of Tregalizumab to all CD4^+^ cells is impacted by Trx1. Trx1 alone at a concentration of 5 μM was able to significantly decrease Tregalizumab binding, but much stronger effects were observed using the physiological Trx1 system, including TrxR and NADPH, allowing for Trx1 recycling. Although the Trx1 concentration exceeds the published levels in plasma or synovial fluid of 1–5 nM,^43^ the local Trx1 concentration at the cell surface of T cells is described to be higher. This might result from the expression and secretion of Trx1 which is noticeably enhanced by some cell types such as dendritic cells and macrophages after activation. Gromer *et al.*^34^ even reported Trx1 tissue concentrations between 1 and 20 μM and calculated intracellular levels of TrxR of 1 μM, illustrating physiological relevance of the used Trx1 concentration.

**Figure 6:**
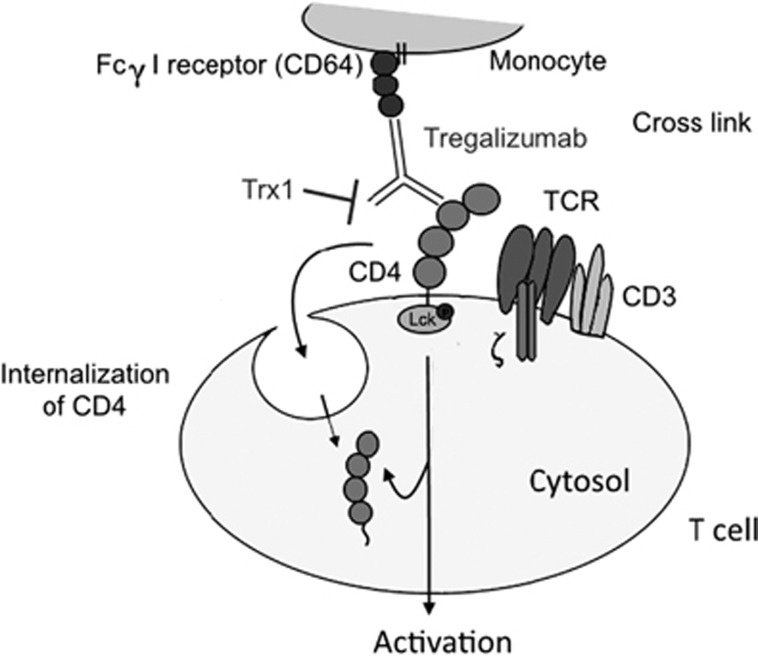
Trx1 inhibits Tregalizumab-mediated signalling via p56*^Lck^*. Trx1 specifically reduces the CD4 disulphide bond in D2. This leads to a diminished binding of Tregalizumab to the CD4 receptor and therefore to a declined signal transduction via p56*^Lck^*.

Stimulation of the CD4 molecule induces signalling events of the lymphocyte-specific protein tyrosine kinase p56^*Lck*^.^44^ Moreover, phosphorylation of ZAP-70 can be detected after activation of the T-cell receptor–CD3 complex.^44^ Helling *et al.*^30^ reported that binding of Tregalizumab to the T-cell receptor induces a unique signalling in T cells via the CD4 molecule. To assess this unique T-cell signalling pathway, we focussed on phosphorylation of p56^*Lck*^ and ZAP-70 as the first molecules of the signalling cascade triggered after activation of CD4 and CD3 by the control antibody OKT3, respectively. We demonstrated that Trx1 preincubation resulted in a significant decrease of p56^*Lck*^ phosphorylation, confirming an impact of Trx1 on Tregalizumab-induced signalling. These findings implicate that Trx1 also indirectly influences CD4-initiated signalling events, subsequent CD4 receptor internalization and probably also activation of T cells in general via influencing binding of the antibody to cell surface CD4. Figure 6 summarizes the proposed Trx1 effect on Tregalizumab-mediated signalling. As Treg function depends on a functional T-cell signalling, addition of Trx1 might have an influence on Treg function in particular. Using *in vitro* assays, it was shown that Tregalizumab is able to activate Tregs selectively in contrast to other CD4 antibodies.^30^ By decreasing binding of Tregalizumab to D2 of CD4 and reducing T-cell activation signal by Trx1, this unique phosphorylation signalling is affected and that is supposed to lead to a declined Treg function. However, determination of CD4 modulation as a pharmacodynamic marker confirmed activity of Tregalizumab in all clinical trials.^31^

As elevated Trx1 levels impacted binding of Tregalizumab with CD4 *in vitro*, we determined Trx1 concentrations in RA patients retrospectively to find a correlation between Trx1 levels and efficacy of the antibody. Measurement of Trx1 levels in RA plasma samples did not reveal a significant correlation between Trx1 plasma concentration and efficacy (data not shown). However, Trx1 is secreted by different cell types such as activated monocytes or lymphocytes^15^ and operates at the site of inflammation where those cells are abundant.^45, 46, 47^ As only Trx1 plasma levels could be analysed, local Trx1 concentrations at the Tregalizumab-binding site on T cells can be much higher. Therefore, high local Trx1 levels maybe present on the cell surface and cannot be detected in the plasma. In addition, it was shown that Trx1 expression and secretion is higher in human Tregs compared with other CD4-postive T cells.^48^ As the RA synovium is enriched with CD4^+^ CD25^+^ Tregs,^49^ a Trx1 secretion by these cells might enhance the survival of autoreactive synovial fluid T cells owing to inhibition of apoptosis by Trx1. This potentially results in aggravation of RA. As Tregalizumab interacts with CD4^+^ CD25^+^ Tregs,^31^ which secrete high amounts of Trx1, it can be speculated that Trx1 might also negatively impact the binding and signalling of Tregalizumab in the synovium of RA patients. So the Trx1 influence on the Tregalizumab activity should be taken into account in other clinical indications of this remarkable therapeutic antibody.

In summary, we hypothesized that elevated Trx1 levels in RA patients are a reason for the observed diminished CD4 downmodulation after Tregalizumab administration compared with healthy subjects. This hypothesis predicts decreased binding of Tregalizumab to CD4 in RA patients owing to higher Trx1 concentrations. And this is exactly what was observed in a retrospective analysis of Biotest clinical studies 979 and 985: relative binding of Tregalizumab to CD4 was significant lower in RA patients compared with healthy subjects, whereas the CD4 expression was nearly comparable in both populations. This finding emphasizes that Tregalizumab's binding is impaired in a particular way in RA patients. As Tregalizumab's target molecule is also a favoured target for T-cell pharmacological regulation in general, for example, by depleting CD4^+^ cells or downmodulating the CD4 receptor, high levels of Trx1 can potentially impact other anti-CD4 antibodies, which bind in close proximity to the D2 disulphide bridge.

## Methods

### Thioredoxin-1

Recombinant human thioredoxins (wild-type Trx1 and inactive mutant Trx1-SS) were provided by the Redox Regulation group at the German Cancer Research Center (DKFZ), DKFZ-ZMBH Alliance, Heidelberg, Germany.

### Preparation of Trx1

Trx1 was incubated with 5 mM DTT (GE-Healthcare, Freiburg, Germany) to reduce all molecules. Prior to experiments, residual DTT was removed using Zeba Spin desalting columns (Thermo Fisher Scientific, Waltham, MA, USA) according to the manufacturer's instructions.

### Blood and plasma

Blood was obtained from healthy blood donors from the blood donation center (Bio-Rad, Dreieich, Germany) and used within 2 h after blood collection.

### Cell culture

Human T-cell leukaemia cells (HPB-ALL) (DSMZ, Braunschweig, Germany) were cultured at 37 °C, in an atmosphere of 5% CO_2_ in Gibco RPMI 1640 medium (Thermo Fisher Scientific) supplemented with 2 mM L-glutamine (Lonza, Basel, Switzerland) and 20% fetal calf serum (FCS, GE-Healthcare, Freiburg, Germany). Cells were spit twice a week and tested for mycoplasma.

### Isolation of PBMCs

Ficoll-Hypaque density-gradient separation was used to obtain PBMCs from citrated blood from healthy subjects. Cells were incubated overnight at 37 °C and 5% CO_2_ in Gibco RPMI 1640 medium (supplemented with 2 mM L-glutamine and 10% FCS). Cells were harvested by centrifugation for 10 min at 220 *g* at room temperature.

### SDS-PAGE (sodium dodecyl sulphate-polyacrylamide gel electrophoresis) and silver staining

SDS-PAGE was performed using a NuPAGE X Cell Sure Lock mini-Cell system (Thermo Fisher Scientific). If possible, samples were diluted to 1 mg ml^−1^ with distilled water. For analysis, non-reduced SDS-PAGE samples were prepared as follows: one part *N*-ethylmaleimide (400 mM) (Alfa Aesar, Karlsruhe, Germany) was mixed with one part lithium dodecyl sulfate sample buffer 4 × (Thermo Fisher Scientific) (NR mixture). The sample was diluted at a ratio of 1:2 with the NR mixture and denaturized by heating for 30 s at 70 °C.

In all, 5–25 μl of samples were pipetted into a ready-to-use NuPAGE Novex 4–12% Bis–Tris gel and NuPAGE Novex 10% Bis-Tris gel (both gels were purchased from Thermo Fisher Scientific). The gel was run for 50 min at 200 V. Molecular weight was determined by SeeBlue Plus 2 Prestained Standard (Thermo Fisher Scientific).

Silver staining was performed using the Silver Stain Plus Kit (Bio-Rad Laboratories, Hercules, CA, USA) according to the manufacturer's instructions.

The gel was incubated for 30 min at 20 r.p.m. in fixation buffer (37% V/V methanol, 7% V/V acetic acid in distilled water) subsequently after electrophoresis to fix the proteins. The gel was washed two times with distilled water for 20 min at 20 r.p.m. on the laboratory flat shaker before staining with silver staining solution. Staining was performed for 5–15 min. The staining procedure was stopped using 5% acetic acid (Merck, Darmstadt, Germany) in distilled water and the gel was washed again for three times for 10 min with distilled water. The gel was scanned using an Epson Perfection V700 Photo scanner (SEIKO Epson CORPORATION, Suwa, Japan).

### Electrochemiluminescence enzyme-linked immunosorbent assay

Ninety-six-well standard microtiter plates (MSD, Rockville, MD, USA) were coated with 50 μl recombinant human soluble (rh sCD4)/carrier free (2 μg ml^−1^) (R&D System, Minneapolis, MN, USA) and incubated for 20±4 h at 2–8 °C.

After the coating procedure, the plates were washed with a plate washer (Tecan, Crailsheim, Germany) three times using 300 μl per well washing buffer (TBST). Afterwards, plates were tapped dry.

A total of 200 μl per well blocking buffer (2% BSA (Sigma Aldrich) in Dulbecco's phosphate-buffered saline (D-PBS, Thermo Fisher Scientific)) was incubated for 75±15 min at room temperature followed by three washing steps with 300 μl per well washing buffer.

Overall, 20 μM of Trx1 were added and incubated for 30±15 min at room temperature.

Before addition of biotinylated Tregalizumab, the plate was washed three times (Biotinylation of Tregalizumab was performed using EZ-Link NHS-SS-Biotin (Thermo Fisher Scientific) according to the manufacturer's instructions).

Solutions were mixed for 90±10 min using a plate shaker (500–700 r.p.m.). A total of 50 μl per well streptavidin-sulfo-TAG (1 μg ml^−1^) (MSD) were added and incubated for 60±15 min at 500–700 r.p.m. and room temperature. After an additional washing step, 150 μl per well Read Buffer T (MSD) (diluted in a ratio 1:2 with distilled water) were added to the samples and shook for 2 min at 500–700 r.p.m. before measurement. Results were obtained using a Sector Imager 6000 (MSD).

### Flow cytometry

Antibodies were titrated for optimal staining prior to use. Labelling of Tregalizumab with APC was performed using an AnaTag APC Labelling Kit (AnaSpec, Seraing, Belgium) according to the manufacturer's instructions. Fluorescein isothiocyanate (FITC)-labelling agent was purchased from Thermo Fisher Scientific, USA. The phycoerythrin (PE)-labelled anti-CD4 antibody OKT4 was purchased from BioLegend (San Diego, CA, USA; order number: 317409). Results were obtained using a four-colour FACS Calibur (BD, Heidelberg, Germany) with the BD CellQuest Software (BD, Heidelberg, Germany).

### Tregalizumab binding to cellular CD4

For binding experiments of Tregalizumab to cells, HPB-ALL cells as well as PBMCs were used. PBMCs were isolated from citrated blood according to the Ficoll-Hypaque density separation protocol as described above.

In all, 1 × 10^5^ cells were incubated with 1 mM NADPH (AppliChem, Darmstadt, Germany), 150 nM thioredoxin reductase (Sigma Aldrich, Buchs, Switzerland) and 5 μM Trx1 in a 96-well U-bottom plate at 37 °C and 5% CO_2_ for 15±2 h overnight. The redox inactive mutant thioredoxin Trx1-SS served as a control. Here, the active site residues Cys 32 and Cys 35 were substituted by serine, and the additional non-active cysteines were replaced by alanine. PBMCs were incubated in RPMI 1640+10% FCS+2 mM L-glutamine; HPB-ALL cells in Dulbecco's phosphate-buffered saline (D-PBS). For assessment of GSH impact on CD4, PBMC cells were pretreated with 10 mM reduced GSH (Sigma Aldrich) and incubated overnight. Cells were centrifuged at 220 *g* for 10 min at room temperature and washed three times using D-PBS.

PBMCs were stained with fluorochrome-labelled antibodies against CD3 (anti-CD3 PerCP, (order number: 345766, BD Biosciences, Heidelberg Germany)) and CD4 (BT-061-APC; Biotest AG, Dreieich, Germany).

Monocytes were gated according to their size and granularity (forward scatter/side scatter (FSC/SSC)). MFI of anti-CD64 PE (order number: 558592, BD Biosciences, Heidelberg Germany) was analysed by means of flow cytometry (BD FACSCalibur).

HPB-ALL cells were stained with BT-061-APC. Mean fluorescence was determined by flow cytometry (BD FACSCalibur).

### Transfection experiments

U266B1 cells were transfected via electroporation using Amaxa Cell Line optimizing Solution Box (Lonza). DNA of CD4 and mutated CD4 was generated by the GeneArt Gene Synthesis service (Thermo Fisher Scientific). In all, 675 μl SF solution was mixed with 150 μl supplement. A total of 5 × 10^5^ cells were centrifuged for 10 min at 250 *g* and room temperature. Pellet was resuspended in 20 μl supplemented SF solution. 4 μg DNA were added to the cell suspension, which was afterwards transferred into a 16-well Nucleocuvette Strip (Lonza). An eGFP (pmaxGFP) served as positive control.

Transfection was performed by Nucleofector (Lonza) applying programme no. DY-100. Owing to regeneration reasons, cells were diluted 1:5 with prewarmed RPMI 1640 medium after the transfection procedure and incubated for 10 min at 37 °C and 5% CO_2_. Afterwards, cells were transferred into a 24-well culture plate and incubated in 500 μl RPMI 1640+10% FCS+2 mM L-glutamine.

### Analysis of signalling of CD4^±^ cells by phosphorylation of proteins

Intracellular phosphorylation of lymphocyte-specific protein tyrosine kinase p56^*Lck*^ and ZAP-70 after stimulation with Tregalizumab or OKT3 was determined using fluorochrome-labelled antibodies (Alexa Fluor 647 anti-Lck (pY505, order number: 558577, PE anti-Zap70 (Y319)/Syk (Y352), order number: 557881, all BD Biosciences, San Jose, CA, USA).

For this purpose, CD4^+^ cells were isolated from human PBMCs via Dynabeads Untouched Human CD4 T Cells Kit (Thermo Fisher Scientific) according to the manufacturer's instructions. A total of 1 × 10^5^ cells were incubated with 5 μM Trx1, 150 nM thioredoxin reductase and 1 mM NADPH overnight for 15±2 h at 37 °C and 5% CO_2_. Cells were washed three times with D-PBS and resuspended in RPMI 1640 enriched with 10% FCS and 2 mM L-glutamine. Tregalizumab (1 μg ml^−1^, Biotest AG) and OKT3 (1 μg ml^−1^, order number: 317304, BioLegend), respectively were added and incubated on a plate shaker (300 r.p.m.) for 30 min at room temperature. Afterwards, cells were washed with D-PBS/3% FCS.

Cells stimulated with Tregalizumab were cross-linked with 20 μg ml^−1^ polyclonal anti-human IgG (ordner number: H10300, Thermo Fisher Scientific), and OKT3-stimulated cells were cross-linked with 10 μg ml^−1^ of anti-murine IgG (order number: 31188, Thermo Fisher Scientific) for 10 min at 37 °C and 5% CO_2_. Prewarmed 100 μl fixation buffer (BD Biosciences, Heidelberg, Germany) was added to each sample, and the cells were incubated for 10 min at 37 °C and 5% CO_2_. After washing with D-PBS/3% FCS, cells were treated with cold Perm Buffer III (BD Biosciences, Heidelberg, Germany), incubated for 30 min on ice and washed again. Cells were stained for 30 min on ice. Afterwards, cells were washed and resuspended in 200 μl D-PBS/3% FCS. MFI was determined using a FACSCanto (FACSDiva Software, BD, Heidelberg, Germany).

MFI of the measured value was divided by the MFI of the untreated control referring to the fold induction.

The inactive mutant Trx1-SS served as a control. Trx1-treated cells were compared with the untreated sample.

### Gating strategies

For PBMCs, the CD4 binding was determined by gating on lymphocytes according to cell size and granularity (FSC/SSC) and then within the gate on CD3^+^ BT-061^+^ cells. The monocyte population was identified by gating of cell size and granularity (FSC/SSC). Within the gate, the fluorescence of PE-labelled anti-CD64 (BD Biosciences, Heidelberg, Germany) was analysed.

For HPB-ALL cells, the CD4 binding was determined by gating on lymphocytes according to cell size and granularity (FSC/SSC) and then within the gate on APC-labelled BT-061^+^ cells.

CD4 binding of transfected U266B1 cells was determined by gating according to cell size and granularity (FSC/SSC) and then within the gate on CD4^+^ cells (APC-labelled BT-061^+^ cells and PE-labelled OKT4^+^ cells).

### Evaluation of clinical data from Biotest studies 979 and 985

Binding of Tregalizumab FITC and anti-CD4 (SK3) PE to lymphocytes was measured using flow cytometry (Biotest study 979, EudraCT: 2010-018485-24, Biotest study 985, EudraCT Number: 2011-004956-20).

Resulting MFI values of gated CD4^+^ T cells (SK3 PE (order number: 347327, BD Biosciences, Heidelberg, Germany) and BT-061 FITC, Biotest) were divided by MFI values of CD4^−^ T cells referring to the ratio of [SK3^+^/CD4^−^] and [BT-061^+^/CD4^−^], respectively. Relative binding of Tregalizumab in relation to total CD4 on the cell surface of T cells was determined by dividing [BT-061^+^/CD4^−^] by [SK3^+^/CD4^−^].

All clinical studies were carried out in accordance with the international guidelines on Good Clinical Practice (ICH-GCP) and in compliance with applicable national regulations. It is confirmed that the studies were carried out and documented in accordance with the corresponding study protocol accepted by regulatory authorities and ethic committees. A signed informed consent was obtained from all subjects.

### Statistical analysis

All data are described as mean±s.d. Normal distribution of data was checked using a Kolmogorov–Smirnov test or a Shapiro–Wilk test. Accordingly, the significance was calculated using unpaired Student's *t*-test or Mann–Whitney test or paired *t*-test or Wilcoxon matched-pairs signed-rank test.

*P*-values were assessed using Prism 6.02 (GraphPad Software, La Jolla, CA, USA). Significance is shown as *P*-values: *P<0.05, **P<0.01, ***P<0.001, ****P<0.0001.
