# Supplementary material for: High thioredoxin-1 levels in rheumatoid arthritis patients diminish binding and signalling of the monoclonal antibody Tregalizumab
Source: Clin Transl Immunology. 2016 Dec 23;5(12):e121–. doi: 10.1038/cti.2016.69 (PMC5192061; doi:10.1038/cti.2016.69)
Supplement: Supplementary Figure 1 [file cti201669x1.pdf]

Supplementary Figure 1

Recombinant human soluble CD4 (rh sCD4) is specifically reduced by Trx1-CC or by the Trx1-CC/Trx reductase system. After treatment, an additional rh sCD4 band appeared, representing reduced rh sCD4 with a lower mobility in the gel.

A

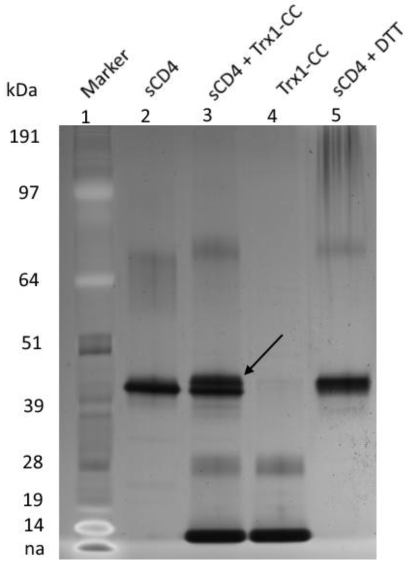

B

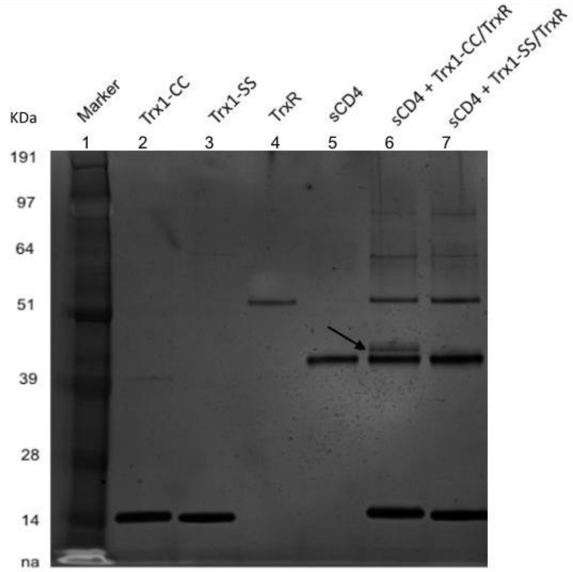

Supplementary Figure 1: Trx1 reduces recombinant human soluble CD4
(**A**) A non-reducing SDS-PAGE (4-12% Bis-Tris gel) was performed to analyze the influence of Trx1 on recombinant human soluble CD4. Silver staining was used to visualize the proteins. 62.5 ng rh sCD4 (~ 40 kDa) was pre-incubated with 2.5 µM Trx1-CC (~ 12 kDa) (lane 3) or 25 mM DTT (lane 5). Trx1 (lane 4) and rh sCD4 (lane 2) were used as controls. The arrow indicates the additional band of reduced rh sCD4.
(**B**) Impact of the Trx1 system, including thioredoxin reductase and NADPH, on rh sCD4. The proteins on the non-reducing SDS-PAGE (10% Bis-Tris gel) were visualised by silver staining. Hexahistidine-SBP-tagged Trx1-CC and the inactive mutant Trx1-SS have a molecular weight of approximately 17 kDa (lane 2 and 3). Thioredoxin reductase exhibited a molecular weight of 55 kDa (lane 4) and rh sCD4 of approximately 40 kDa (lane 5). Trx1-CC/TrxR pre-incubation caused an additional band with a higher molecular weight, representing reduced rh sCD4 (lane 6, indicated by arrow).
